# A Preclinical State of Graves' Ophthalmopathy Characterized by Hypoxia of T-cells Identified via Multiomics Analysis

**DOI:** 10.1210/clinem/dgaf230

**Published:** 2025-04-11

**Authors:** Meng Zhang, Xin Qi, Xingchen Zhou, Yufeng Liu, Mingqian He, Jingya Wang, Ling Wang, Ziyi Chen, Simo Li, Yu Chen, Yushi Sun, Hui Guo, Jin Yang, Bingyin Shi, Yue Wang

**Affiliations:** Department of Endocrinology, The First Affiliated Hospital of Xi’an Jiaotong University, Xi’an 710061, China; Precision Medicine Center, The First Affiliated Hospital of Xi'an Jiaotong University, Xi'an 710061, China; Department of Endocrinology, The First Affiliated Hospital of Xi’an Jiaotong University, Xi’an 710061, China; Biobank, The First Affiliated Hospital of Xi’an Jiaotong University, Xi’an 710061, China; Department of Endocrinology, The First Affiliated Hospital of Xi’an Jiaotong University, Xi’an 710061, China; Department of Gastroenterology, Xi’an Children's Hospital, Shaanxi Research Institute for Pediatric Diseases, The Affiliated Children's Hospital of Xi’an Jiaotong University and National Regional Medical Center for Children (Northwest), Xi’an 710003, China; Department of Endocrinology, The First Affiliated Hospital of Xi’an Jiaotong University, Xi’an 710061, China; Lineberger Comprehensive Cancer Center, University of North Carolina at Chapel Hill, Chapel Hill, NC 27599, USA; Department of Endocrinology, The First Affiliated Hospital of Xi’an Jiaotong University, Xi’an 710061, China; Department of Endocrinology, The First Affiliated Hospital of Xi’an Jiaotong University, Xi’an 710061, China; Department of Endocrinology, The First Affiliated Hospital of Xi’an Jiaotong University, Xi’an 710061, China; Department of Endocrinology, The First Affiliated Hospital of Xi’an Jiaotong University, Xi’an 710061, China; Precision Medicine Center, The First Affiliated Hospital of Xi'an Jiaotong University, Xi'an 710061, China; Department of Medical Oncology, The First Affiliated Hospital of Xi’an Jiaotong University, Xi’an 710061, China; Department of Endocrinology, The First Affiliated Hospital of Xi’an Jiaotong University, Xi’an 710061, China; Department of Endocrinology, The First Affiliated Hospital of Xi’an Jiaotong University, Xi’an 710061, China

**Keywords:** Graves' ophthalmopathy, preclinical state, T-cells, hypoxia, multiomics analysis

## Abstract

**Context:**

A preclinical state of Graves' ophthalmopathy (pre-GO) exists during the progression from Graves' hyperthyroidism (GH) to GO.

**Objective:**

To distinguish the pre-GO state and identify key pathways of T-cell immunity.

**Methods:**

Twenty-four GH (without ophthalmopathy within 6-month follow-up), 10 pre-GO (ophthalmopathy occurred within 6-month follow-up), and 21 GO patients were enrolled, and the transcription and DNA methylation profiles of peripheral blood mononuclear cells were generated. The differentially expressed genes (DEGs), differentially methylated CpG sites (DMCs), and differentially methylated genes (DMGs) were identified. Cluster analysis, functional analysis, and data integration analysis using latent components (DIABLO) were performed to distinguish pre-GO and identify key pathways. Flow cytometry was performed for in vitro verification.

**Results:**

In total, 731, 1214, and 372 DEGs and 1583, 277, and 555 DMCs were detected via pairwise comparisons of GH vs GO, pre-GO vs GO, and GH vs pre-GO, respectively. DIABLO accurately discriminated the pre-GO state via 17 DMC and 11 DEG features ( receiver operating characteristic = 0.9975 and 0.9407, respectively). The functional analysis revealed that the DMGs and DEGs were enriched in T-cell differentiation pathways and related cytokine pathways, respectively. Further cluster analysis revealed a cluster of pre-GO-specific DEGs enriched in the hypoxia pathway. Flow cytometry confirmed that hypoxia promoted Th1, Th17, and antigen-specific CD4+ cytotoxic T-cell differentiation.

**Conclusion:**

The pre-GO state was identified from GH and GO and characterized by upregulation of the hypoxia pathway that may promote effector CD4+ T-cells differentiation. These findings provide new insight into the pathogenesis and prevention of GO.

Graves' hyperthyroidism (GH), an autoimmune disorder, is the main cause of hyperthyroidism in adults (1). Graves' ophthalmopathy (GO) is the most common extrathyroidal manifestation of GH, occurring in up to 50% of GH cases (2). Severe GO, characterized by disfiguring and functionally impactful corneal breakdown or optic neuropathy, occurs in 3% to 5% of GH patients and substantially decreases patient quality of life (3). The pathological changes associated with GO are orbital inflammation, adipogenesis, fibrosis, and enlargement of the extraocular muscles, which lead to irreversible tissue remodeling (4). Glucocorticoids have been the mainstay GO treatment since the last century, but the effect of steroid monotherapy remains suboptimal (5). Recently, mycophenolate, rituximab, tocilizumab, teprotumumab, and rapamycin have been reported to achieve some benefits in clinical trials (6-9). However, as the therapeutic options for GO are currently highly limited and fail to achieve satisfactory results, development of novel strategies for the management of GO is urgently needed (10).

In the clinic, orbital symptoms often occur after the onset of hyperthyroidism (predominantly within 18 months), which is reportedly related to smoking and dyslipidemia (2, 11-13). The progression from GH to GO has been described as a “double whammy,” recapitulating thyroid dysfunction and progressing to the development of orbital complications over time (14). Recently, a pre-GO state in GH patients was identified via a T-cell receptor-based model, in which GH patients progressed to GO within approximately 6.5 months (15). Preclinical mouse models confirmed the existence of a pre-GO state of GH with immune changes (10). CD4+ T-cells are assumed to be involved in the immune changes in pre-GO, as these cells play an important role in initiating and perpetuating orbital inflammation in GO (16). Clinical reports have shown that different CD4+ T-cell effector subsets are predominant in the peripheral blood of GH and GO patients, which also indicates the evolution of CD4+ T-cells during the progression from GH to GO (17, 18). Thus, identifying the characteristics of T-cells in the pre-GO state is imperative for understanding the underlying pathogenesis and preventing progression to GO.

RNA sequencing (RNA-seq) can provide transcriptomic information, and DNA methylation is an important epigenetic modification that connects genetics, the regulation of gene expression, and environmental risk factors (19). Several reports have shown that T-cells exhibit abnormal changes in transcript and DNA methylation profiles during GO progression. Single-cell transcriptomic analysis revealed GO-specific CD4+ cytotoxic T-cells marked with granzyme B (GZMB) (20). Epigenetics may also impact the occurrence of GO through pathways involved in cell killing, cytokine production, and the immune response (21). Therefore, integrated analysis of mRNA and DNA methylation profiles may provide a more comprehensive understanding of the functional and biological characteristics of T-cells in this disease.

In the present study, we recruited GO, GH, and pre-GO patients, who were identified at the 6-month follow-up (Fig. 1A). RNA-seq and an 850k methylation array were performed on peripheral blood mononuclear cells (PBMCs) to identify the key biological pathways of T-cells in the pre-GO state, which was subsequently verified by in vitro experiments (Fig. 1A and 1B). This information will be highly useful for clarifying the characteristics of the pre-GO state and identifying candidate prophylactic interventions for GO in future research.

**Figure 1.**
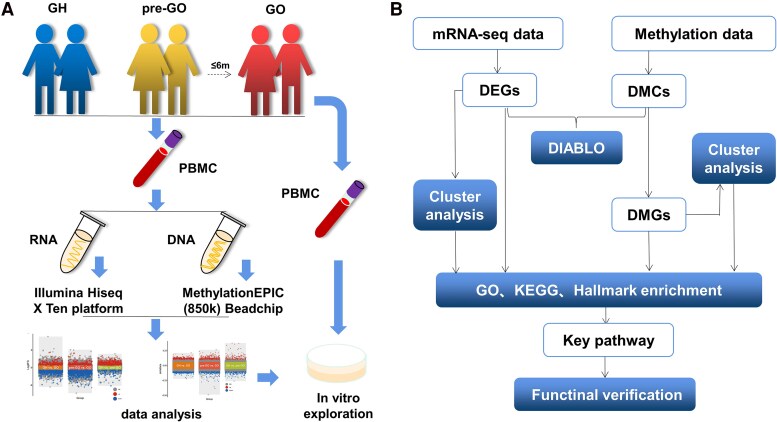
(A) The study design of this study; (B) the detailed analysis used in this analysis.

## Materials and Methods

### Study Subjects

Twenty-four GH, 10 pre-GO, and 21 GO patients were enrolled in this study. The pre-GO patients were patients who were initially diagnosed as hyperthyroidism but progressed to ophthalmopathy by 6-month follow-up, whereas GH patients were patients whose disease was not associated with ophthalmopathy within the 6-month follow-up. All the GO patients were diagnosed by professional clinicians on the basis of their clinical manifestations and orbital computed tomography results. PBMCs were isolated from the blood of patients as described previously (22), after which RNA-seq and DNA methylation array were performed. The detailed characteristics of the participants are shown in Table 1.

**Table 1.** Clinical characteristics of the patients

	All	GH*^a^* (n = 24)	Pre-GO*^b^* (n = 10)	GO (n = 21)	*P*
Age (years)	39.7 ± 1.7	38.0 ± 2.1	37.8 ± 3.5	42.6 ± 3.5	.595
Sex (%)					**.035**
Male	18 (32.7)	6 (25.0)	1 (10.0)	11 (52.4)	
Female	37 (67.3)	18 (75.0)	9 (90.0)	10 (47.6)	
BMI (kg/m^2^)	21.7 ± 0.4	20.8 ± 0.6	20.6 ± 0.7	23.2 ± 0.6	**.004**
Smoking status (%)					**.003**
Smoker	14 (25.5)	4 (16.7)	0 (0)	10 (47.6)	
Nonsmoker	41 (74.5)	20 (83.3)	10 (100)	11 (52.4)	
History of thyroid dysfunction (%)					.186
Hyperthyroidism	53 (96.4)	24 (100)	10 (100)	19 (90.5)	
Euthyroidism	2 (3.6)	0 (0)	0 (0)	2 (9.5)	
Status of thyroid function (%)					**<.001**
Hyperthyroidism	38 (69.1)	21 (87.5)	9 (90)	8 (38.1)	
Euthyroidism	17 (30.9)	3 (12.5)	1 (1)	13 (61.9)	
Positive TPOAb*^c^* (%)	35 (74.5)	17 (85)	5 (38.5)	13 (68.4)	.344
Positive TGAb*^c^* (%)	12 (23.1)	5 (22.7)	1 (10)	6 (30)	.471
Positive TMAb*^c^* (%)	12 (23.1)	5 (22.7)	1 (10)	6 (30)	.471
Positive TRAb*^c^* (%)	29 (63)	14 (66.7)	6 (75)	9 (52.9)	.508
CAS*^d^*	/	/	2 (1, 3)	2 (2, 3)	.528*^e^*
NOSPECS*^d^*	/	/	2 (1, 3)	3 (2, 4)	**.027** *^e^*

Bold values indicate significant differences (*P* < .05).

Abbreviations: BMI, body mass index; CAS, clinical activity score; GH, Graves' hyperthyroidism; GO, Graves' ophthalmopathy; TGAb, thyroglobulin antibodies; TMAb, thyroid microsomal antibodies; TPOAb, thyroid peroxidase antibodies; TRAb, thyroid receptor antibodies.

^*a*^GH patients diagnosed with GH and no ophthalmopathy were observed at the 6-month follow-up.

^*b*^Pre-GO patients who were initially diagnosed with GH but progressed to GO by the 6-month follow-up.

^*c*^Missing values in less than 20% of patients.

^*d*^Data from GO patients were collected at the time of enrollment, and data from pre-GO patients were collected at the time of diagnosis with ophthalmopathy during follow-up.

^*e*^ *P*-value for comparison between the GO group and pre-GO group.

### Ethics

All patients were recruited from the Department of Endocrinology, The First Affiliated Hospital of Xi’an Jiaotong University. This study was approved by the Ethics Committee of The First Affiliated Hospital of Xi’an Jiaotong University (no. XJTUIAF2022LSK-383). All participants provided informed consent.

### PBMC Culture and Stimulation In Vitro

The PBMCs were cultured as previously described (22). For in vitro nonspecific stimulation, purified anti-human CD3 antibodies (OKT3, 317325, Biolegend, USA) were coated at 10 μg/mL overnight at 4 °C, and purified anti-human CD28 antibodies (28.2, 302934, Biolegend) were added to the medium at a final concentration of 2 μg/mL. For in vitro antigen-specific stimulation, recombinant human TSH receptor (TSHR) protein was added at a final concentration of 25 μg/mL, as TSHR serves as the primary autoantigen in GO and GH (20). The PBMCs were cultured in a 24-well plate at a concentration of 1.5 × 10^6^ cells/mL for 48 hours at different O^2^ concentrations (21%, 10%, and 1%) via a PhO2x Box (Baker, UK).

### Flow Cytometry

PBMCs were suspended in staining buffer (eBioscience) at a final concentration of 1 × 10^-7^ cells/mL and incubated with fluorescein isothiocyanate-conjugated anti-human CD3 (BD Biosciences Cat# 555339, RRID:AB_395745) and allophycocyanin-conjugated anti-human CD8 (BD Biosciences Cat# 555369, RRID:AB_398595) antibodies for extracellular staining. Then the cells were fixed and permeabilized with the transcription factor buffer set (562574, BD) and incubated with phycoerythrin-conjugated anti-human interferon (IFN)-γ (BioLegend Cat# 502508, RRID:AB_315233), Brilliant Violet 421-conjugated anti-human IL-17a (BioLegend Cat# 512321, RRID:AB_10899566), and phycoerythrin/Cyanine7-conjugated anti-human/mouse GZMB (BioLegend Cat# 372214, RRID:AB_2728381) antibodies for intracellular staining. Flow cytometric analysis was performed via a Novocyte Advanteon Flow cytometer (Agilent Technologies, USA), and the CD3 + CD8− cells were considered as CD4+ T-cells. The data were analyzed via Novoexpress (Agilent Technologies).

### RNA-seq, Differential Expression Analysis, and Functional Analysis

Fifty-five samples derived from 24 GH, 10 pre-GO, and 21 GO patients were used for RNA-seq. mRNA expression profiles were obtained from the Illumina HiSeq 3000 platform. After quality control, we used the “DESeq2” R package to perform differential expression analysis (23). Three groups were compared in this analysis: GH vs GO, GH vs pre-GO, and pre-GO vs GO. The genes with absolute log2 fold change (FC) ≥ 0.58 and *P*-value <.05 were defined as significantly differentially expressed genes (DEGs). For significant DEGs in the 3 compared groups, gene set enrichment analysis, including Gene Ontology (GO), Kyoto Encyclopedia of Genes and Genomes (KEGG), and hallmark pathway, was performed via the R package “clusterProfiler” (24). The reference gene sets were in the Molecular Signatures Database, and the pathways with *P-*value <.05 were selected as the significant pathways.

In addition, we used the R package “mfuzz” to obtain the DEGs expression patterns associated with 3 different disease severity (25). For the DEGs in different clusters, we performed functional analysis, including GO, KEGG, and hallmark, using the R package “clusterProfiler” (24).

### DNA Methylation, Differentially Methylation Analysis, and Functional Analysis

Fifty-four samples derived from 24 GH, 9 pre-GO, and 21 GO patients were used for DNA methylation. Genome-wide DNA methylation was evaluated via the Illumina Infinium HumanMethylation 850 K BeadChip (Illumina Inc., USA) according to the manufacturer's instructions. The array data (.IDAT files) were analyzed via the ChAMP package in R to derive the methylation level. The methylation status of all the probes was denoted as a β value ranging from 0 to 1, which represents the ratio of the methylated probe intensity to the overall probe intensity (sum of the methylated and unmethylated probe intensities plus a constant α, where α = 100).

According to the compared groups, the differentially methylated CpG sites (DMCs) and regions were analyzed via the R package “limma” and DMRcate (v1.20.0), respectively. DMCs with |Δβ| ≥ 0.10 and *P*-value ≤.05 were considered as significant DMCs. A CpG was deemed hypermethylated if Δβ ≥ 0.10 or hypomethylated if Δβ≤−0.10. The average β value of promoters and CpG islands was compared between the 3 groups. According to the differentially methylated genes (DMGs) of DMCs and differentially methylated CpG regions, GO, KEGG, and hallmark functional analyses were performed via gene set enrichment analysis with R package “clusterProfiler.” A *P*-value <.05 was considered significant.

In addition, the R package “mfuzz” was used to obtain the DMCs expression patterns for 3 different diseases’ severity (25). For the DMGs in different clusters, we performed functional analysis, including Gene Ontology, KEGG, and hallmark, via the R package “clusterProfiler” (24).

### Integrated Analysis of mRNA Expression and DNA Methylation Profiles

We integrated RNA expression and DNA methylation profiles to investigate their interplay during the progression from GH to GO. Initially, we identified common functions between pathways of DEGs and DMGs to discover meaningful associations with GH and GO. We subsequently utilized the Data Integration Analysis and Biomarker discovery using Latent variable approaches for Omics studies (DIABLO) via the R package “mixOmics” (26). DIABLO, a multivariate integrative classification method, usually facilitates the identification of correlated or coexpressed variables from heterogeneous datasets. We employed the N-integration supervised Sparse PLS-DA approach for variable selection, determining latent structures composed of highly correlated variables across both datasets (27). The optimal number of components and variables was chosen via cross-validation with 5 folds and 10 repetitions through the perf function of the mixOmics package. Then, we applied the block.splsda function to the selected variables to evaluate correlations between DEGs and DMGs expression, differentiating among GH, pre-GO, and GO statuses. Finally, the DEGs and DMCs identified by mixOmics were employed to assess diagnostic performance via receiver operating characteristic (ROC) curves and area under the curve. Statistical significance was defined at *P* ≤ .05.

### Statistics

Statistical analysis was performed using SPSS 25.0 software. For continuous variables, when the data were normally distributed and showed homogeneity of variance, data were expressed as mean ± SD, with Student's *t*-tests for 2 groups and ANOVA tests for 3 groups used to calculate statistical significance; otherwise, data were reported as median and interquartile range, with the Mann–Whitney U-test for 2 groups and Kruskal–Wallis tests for 3 groups used to calculate statistical significance. For categorical variables, data were expressed as frequency rates and percentages, and the chi-squared test was used for group comparisons. For the in vitro experiment, 2-tailed, paired samples *t*-tests were used for group comparisons. Unless otherwise noted, *P* < .05 was considered statistically significant.

## Results

### Clinical Characteristics of Patients

We enrolled 24 GH, 10 pre-GO, and 21 GO patients in this study, with pre-GO patients defined as those who were initially diagnosed with GH but had progressed to GO by the 6-month follow-up. Table 1 shows the clinical characteristics of these patients. There were more males and smokers in the GO group (*P* = .035 and *P* = .003), which is consistent with previous reports (11). The GH and pre-GO patients had a lower body mass index (*P* = .004); this result may be related to differences in thyroid function between the groups, and the dyslipidemia reported to be involved in GO pathogenesis as well (12, 13). There were no significant differences among the 3 groups in terms of age, history of thyroid dysfunction, or antibodies.

Ophthalmic assessments included the clinical activity score for disease activity, and the NOSPECS (no TAO sign, only eyelid sign, soft tissue involvement, proptosis, extraocular motility restriction, corneal involvement, sight loss) score for disease severity. The NOSPECS score of pre-GO patients was lower than that of GO patients; this finding may be because the former was detected in time during follow-up without further progression. The clinical activity score did not significantly differ between the 2 groups.

### DEGs and Functional Analysis in Pairwise Comparisons

**Figure 2.**
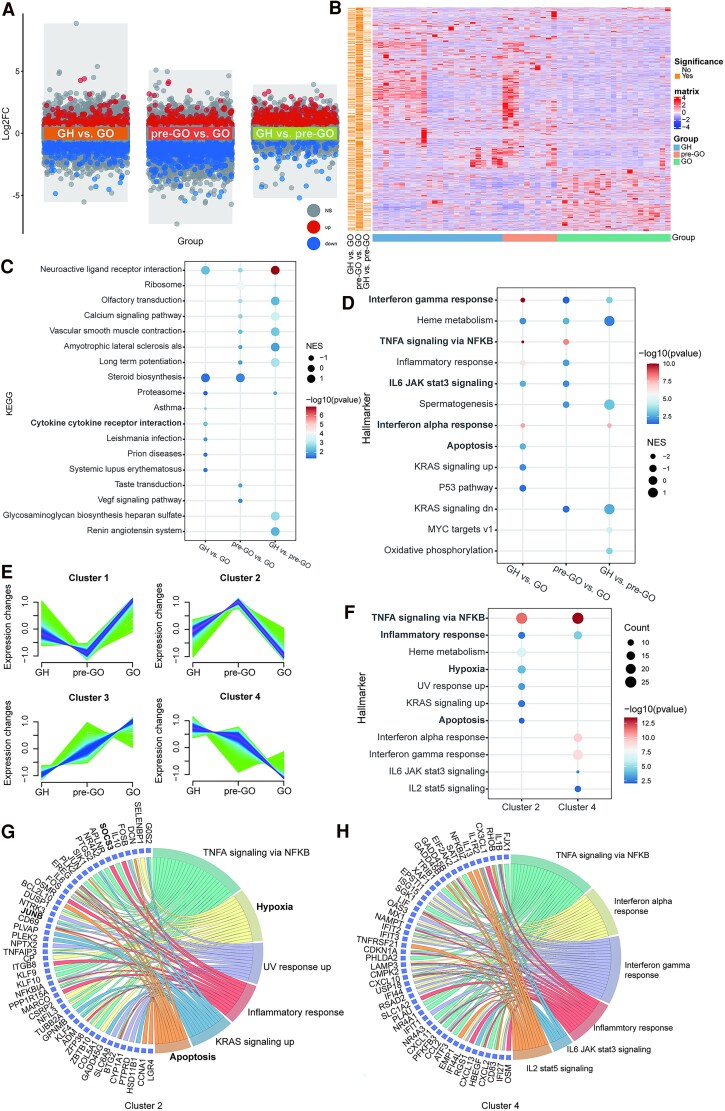
DEGs associated with GH, pre-GO, and GO. (A) The volcano plot of DEGs in GH vs GO, pre-GO vs GO, and GH vs pre-GO. (B) Heatmap of DEGs in GH vs GO, pre-GO vs GO, and GH vs pre-GO. (C) KEGG analysis results of DEGs in GH vs GO, pre-GO vs GO, and GH vs pre-GO. (D) Hallmarker analysis results of DEGs in GH vs GO, pre-GO vs GO, and GH vs pre-GO. (E) Fuzzy c-means clustering identified 4 distinct patterns of gene expression. The x-axis represents 3 disease stages, while the y-axis represents log2-transformed, normalized intensity ratios in each stage. (F) Hallmarker analysis results of genes in 4 patterns. (G) Chord plot of hallmarker analysis results for genes in cluster 2. (H) Chord plot of hallmarker analysis results for genes in cluster 4. Abbreviations: DEG, differentially expressed gene; FC, fold change; GH, Graves' hyperthyroidism; GO, Graves' ophthalmopathy; KEGG, Kyoto Encyclopedia of Genes and Genomes.

Pairwise comparisons of transcriptome sequencing data were performed among the 3 groups because abnormal GO-related T-cell immunity was detected in the pre-GO state (10, 15). Overall, 731, 1214, and 372 DEGs were identified between the GH and GO, pre-GO and GO, and GH and pre-GO groups, respectively (Fig. 2A and 2B, Supplementary Tables S1-S3) (28).

The Gene Ontology and KEGG analyses both revealed that the DEGs between the GH and GO groups were enriched in the inflammatory response and cytokine-mediated signaling pathways (Fig. 2C, Supplementary Fig. S1, Supplementary Tables S4-S9) (28). The hallmark pathway enrichment analysis also revealed that the TNF-α, IFN-α, and IFN-γ pathways and the inflammatory response-related pathway, as well as the apoptosis-related pathway, were enriched in the GH vs GO comparison (Fig. 2D, Supplementary Tables S10-S12) (28).

### Cluster-based DEG Functional Analysis Among the 3 Groups

For further elucidation of the various changes in genes associated with the progression from GH to GO, the DEGs identified here were further divided into 4 clusters (Fig. 2E, Supplementary Table S13) (28), and DEGs in cluster 2 were upregulated in the pre-GO state compared with those in the GH and GO state.

Gene Ontology and KEGG enrichment analyses revealed that the DEGs in cluster 1 and cluster 4 were enriched in cytokine- and chemokine-related signaling pathways, whereas the DEGs in cluster 4 were also enriched in the IL-17 and TNF signaling pathways (Supplementary Fig. S2A–E, Supplementary Tables S14-S21) (28). However, the DEGs in cluster 2 were enriched in pathways related to the hydrogen peroxide catabolic process and oxygen metabolism by GO enrichment and in the IL-17 and TNF signaling pathways by KEGG analysis (Supplementary Fig. S2B and S2E, Supplementary Tables S15 and S19) (28). Hallmark pathway enrichment analysis also revealed that hypoxia, apoptosis, TNF-α, and the inflammatory response pathway were enriched in cluster 2 (Fig. 2F-2H, Supplementary Tables S22 and S23). Thus, considering the pattern of mRNA expression changes shown in cluster 2, the pre-GO state exhibited specific gene expression characteristics that were distinct from those of the GH and GO states, specifically involving hypoxia and apoptosis.

### DMCs, DMGs, and Functional Analysis in Pairwise Comparisons

**Figure 3.**
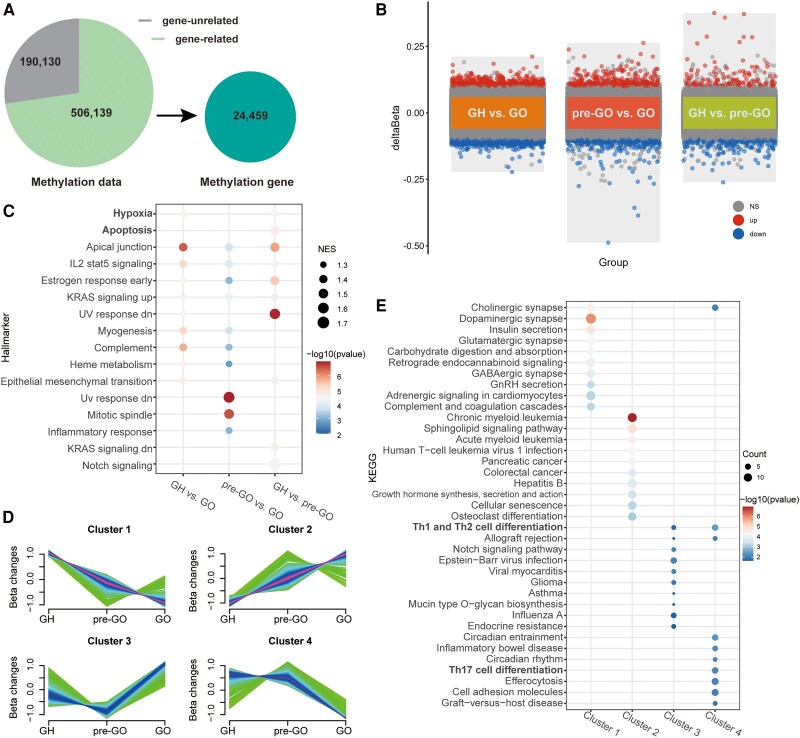
DMCs and DMGs associated with GH, pre-GO, and GO. (A) Total description of CpGs and methylated genes. (B) The volcano plot of DMCs in GH vs GO, pre-GO vs GO, and GH vs pre-GO. (C) Hallmarker analysis results of DMGs in GH vs GO, pre-GO vs GO, and GH vs pre-GO. (D) Fuzzy c-means clustering identified 4 distinct patterns of DMGs expression. The x-axis represents 3 disease stages, while the y-axis represents log2-transformed, normalized intensity ratios in each stage. (E) KEGG analysis results of DMGs in 4 clusters. Abbreviations: DMC, differentially methylated CpG site; DMG, differentially methylated gene; GH, Graves' hyperthyroidism; GO, Graves' ophthalmopathy; KEGG, Kyoto Encyclopedia of Genes and Genomes.

A total of 19 130 gene-unrelated and 506,139 gene-related DNA methylation probes were detected, and the latter were attributed to 24 459 methylated genes (Fig. 3A). Pairwise comparisons revealed 1583, 277, and 555 DMCs between the GH and pre-GO groups, the GH and GO groups, and the GO and pre-GO groups, respectively (Fig. 3B, Supplementary Fig. S3, and Supplementary Tables S24-S26) (28).

The GO functional enrichment analysis revealed that the DMGs in the GH vs GO and GH vs pre-GO comparisons were both enriched in the cell adhesion pathway (Supplementary Fig. S4A-4C, Supplementary Tables S27-S29) (28). The KEGG pathway analysis revealed that the DMGs in the GH vs GO comparison were enriched in the chemokine signaling pathway (Supplementary Fig. S4D, Supplementary Tables S30-S32) (28). Hallmark pathway enrichment analysis revealed that hypoxia- and apoptosis-related genes were enriched in both the GH vs GO and the GH vs pre-GO comparisons (Fig. 3C, Supplementary Tables S33-S35) (28).

### Cluster-based DMG Functional Analysis Among the 3 Groups

A 4-classification cluster analysis for the DMGs was also performed (Fig. 3D, Supplementary Table S36) (28). GO enrichment analysis revealed that the DMGs in both clusters 2 and 3 were enriched in T-cell differentiation and adhesion pathways, and those in cluster 4 were enriched in the regulation of the inflammatory response (Supplementary Fig. S5, Supplementary Tables S37-S40) (28). Similarly, KEGG enrichment analysis revealed enrichment of synapse-related pathways in cluster 1, cellular senescence in cluster 2, Th1/Th2 differentiation in cluster 3, and Th17 and Th1/Th2 differentiation in cluster 4 (Fig. 3E, Supplementary Tables S41-S45). In particular, the pattern of DNA methylation changes in cluster 4 indicates that genes in the Th1, Th2, and Th17 differentiation pathways were hypomethylated during pre-GO to GO progression.

### DIABLO Identifies Multiomic Signatures That Can Discriminate the Pre-GO State

**Figure 4.**
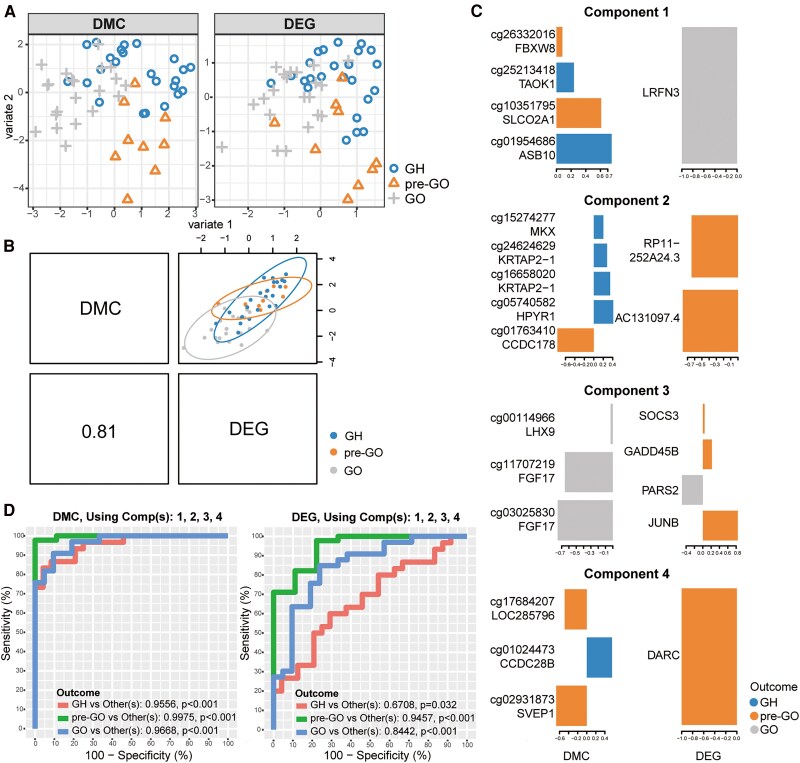
The integration of gene expression and methylated genes through DIABLO. (A) The individuals’ distribution according to the selected variables; (B) the correlation of DMGs and DEGs in component 1; (C) the variables of DMGs and DEGs selected by 4 components; (D) receiver operating characteristic of selected variables in methylated data and gene expression data. Abbreviations: DEG, differentially expressed gene; DIABLO, data integration analysis using latent components; DMG, differentially methylated gene.

DIABLO was used to integrate our mRNA data and DNA methylation data to predict the status of a sample (GH vs pre-GO vs GO). The sample plots of the final DIABLO model in Fig. 4A indicate better discrimination of pre-GO with the DMC data than with the DEG data. Figure 4B shows that the latent components of each omics dataset were highly correlated with each other, highlighting the ability of the DIABLO model to capture good agreement between the datasets.

Four components were selected to distinguish the progression from GH to GO in this study. In total, we discovered 17 DMC and 11 DMG features, including FBXW8 (cg26332016), TAOK1 (cg25213418), JUNB, and SOCS3 (Fig. 4C). Notably, JUNB and SOCS3 were also found among the genes in cluster 2 according to the DEG analysis (Fig. 2G).

The multiomic signature described here was able to successfully classify pre-GO vs others (Fig. 4D). The achieved ROCs were 0.9975 and 0.9407 for the DMPs and mRNAs, respectively.

### Hypoxia and T-cell Differentiation are Key Pathways of the Pre-GO State

T-cells, especially CD4+ T-cells, play important roles in initiating and perpetuating orbital inflammation in GO (16). Thus, we screened for pathways enriched in T-cell- and immunity-associated pathways. DMGs were enriched mainly in T-cell differentiation pathways, and DEGs were enriched mainly in cytokine- and chemotaxis-mediated pathways, which are closely related to T-cell differentiation. In particular, during the pre-GO to GO progression, the genes in the Th1, Th2, and Th17 differentiation pathways were hypomethylated (cluster 4, Fig. 3D and 3E), suggesting that the functions of the genes in the relevant pathways were promoted. These results are consistent with those previous studies, indicating the important role of CD4+ T-cell subset imbalance in GO (17, 29).

Hypoxia was revealed as a biological characteristic of the pre-GO state in patients (Fig. 2E–2H). Many studies have shown that hypoxia governs the differentiation and functions of CD4+ T-cells, especially Th1 and Th17 cells (30-32). In addition, a GO-specific CD4+ cytotoxic T-cell subgroup (CTL) may also be promoted by hypoxia, as a low oxygen level was reported to increase the cytotoxicity of T-cells (20, 33). Thus, given that T-cell differentiation pathways are key functional pathways during the progression of pre-GO to GO, we hypothesize that hypoxia in the pre-GO state may affect the differentiation of CD4+ T-cells, accompanied by changes in cytokines and chemotaxis.

### Hypoxia Promoted Effector CD4+ T-cell Differentiation In Vitro

**Figure 5.**
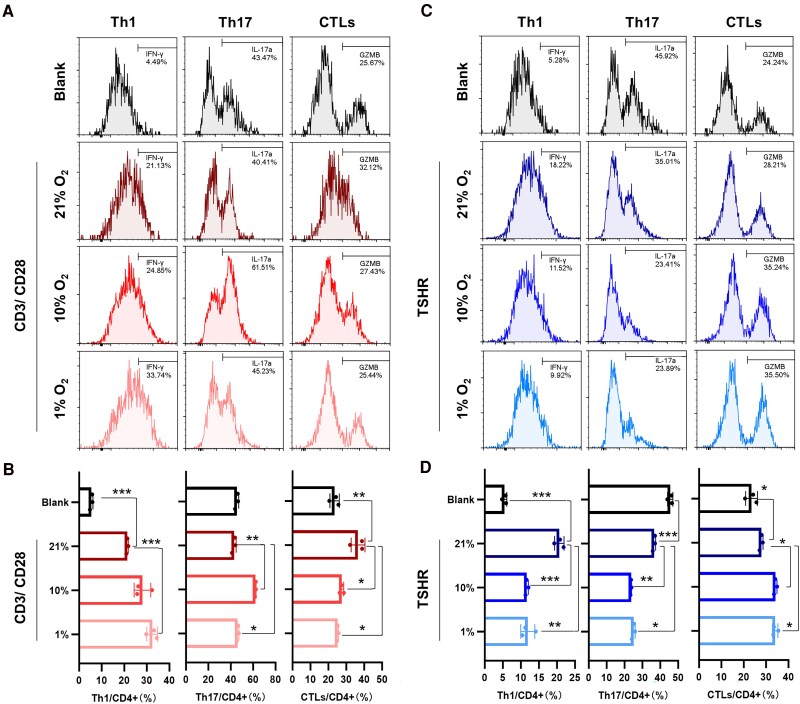
The in vitro effect on hypoxia on T-cell differentiation. (A) and (B) Flow cytometric analysis of the proportions of Th1, Th17, and CD4+ CTLs in PBMCs of GO patients stimulated by CD3/CD28 antibodies with or without hypoxia (n = 3). FACS plots and bar plots are presented. (C) and (D) Flow cytometric analysis of the proportions of Th1, Th17, and CD4+ CTLs in PBMCs GO patients stimulated by TSHR with or without hypoxia (n = 3). FACS plots and bar plots are presented. * *P* < .05, ** *P* < .01, *** *P* < .001. Abbreviations: CTL, CD4+ cytotoxic T-cell; FACS, fluorescence-activated cell sorting; PBMC, peripheral blood mononuclear cell; TSHR, TSH receptor.

To further explore the effect of hypoxia on T-cells, PBMCs from GO patients were isolated and cultured at different O_2_ concentrations (21%, 10%, and 1%) with stimulation by CD3/CD28 mAbs. Compared with control PBMCs, PBMCs stimulated with CD3/CD28 mAbs for 48 hours presented increased Th1 (CD4 + IFN-γ+) and CD4+ CTL (CD4 + GZMB+) proportions but no change in the Th17 (CD4 + IL−17a+) proportion (Fig. 5A and 5B). The 10% O_2_ intervention resulted in an increase in the Th17 proportion, and the 1% O_2_ intervention resulted in increases in both the Th1 proportion and the Th17 proportion. However, the proportion of CD4+ CTLs decreased after 10% and 1% O_2_ intervention.

TSHR is considered the primary autoantigen in GO on the basis of the close temporal correlation of its expression with that of GO and GH, findings from a GO animal model, and the relationship of disease activity with anti-TSHR antibodies (4, 34-36). Thus, we examined cells treated with the TSHR protein to explore the effect of TSHR-specific T-cells under hypoxia (Fig. 5C and 5D). Compared with the control cells, cells stimulated with TSHR for 48 hours presented increased Th1 and CD4+ CTL proportions and a decreased Th17 cell proportion. Both the 10% and 1% interventions resulted in an increase in the CD4+ CTL proportion but a decrease in the Th1 and Th17 proportions.

In conclusion, hypoxia promoted the differentiation of different proinflammatory Th cells, manifested as Th1/Th17 differentiation under CD3/CD28 stimulation and CD4+ CTL differentiation under specific antigen stimulation.

## Discussion

GO pathogenesis is a complex biological process that remains incompletely understood, and current therapies are often accompanied by severe side effects and numerous complications (5). Therefore, prophylactic intervention has become a potential strategy, closely related to the definition and characterization of the incubation period. In the present study, we used integrated analysis of DNA methylation and transcriptome data from PBMCs to distinguish the pre-GO state from the GH and GO states, revealing that it is characterized by promotion of the hypoxia pathway and consequent promotion of effector CD4+ T-cell differentiation. These data confirm the existence of a latent period of GO and provide insights for the development of predictive and preventive interventions for GO.

According to our integrated analysis, the pre-GO state (defined as the 6 months before the onset of ophthalmopathy in patients with GH) significantly differed from the GH and GO states in terms of T-cell immunity (37, 38). Unlike previous studies, which were based on sequencing data alone, our study utilized PBMC DNA methylation and transcriptome data derived from the same blood sample from the same patient. Then, multiomic features were identified via DIABLO, resulting in an ROC area under the curve of 0.9 for the pre-GO state. Considering these findings in the context of the clinical information of these patients, we propose a 6-month incubation period for the progression from GH to GO, which is consistent with the findings of previous clinical and animal studies (10, 15).

T-cell differentiation, cytokines, and chemotaxis were enriched as key functional pathways during the progression of GH to GO. After activation, T-cells may differentiate into various effector phenotypes, including Th1, Th2, or Th17 cells (39). The proportion of Th1 cytokines in GO patients is significantly greater than that in GH patients, and the proportion of Th1 cells and the Th1/Th2 ratio are positively correlated with the clinical activity of GO (17). Several studies have shown that IL-17A levels in the blood and the proportion of Th17 cells in GO patients are greater than those in healthy controls (29, 40-42). In our study, pathways related to Th1 cells and cytokines (IL-2, IFN-γ) and Th17 cells and cytokines (IL-17) were enriched in the progression of GH to GO. Moreover, the enrichment of multiple cytokine and chemotaxis pathways also indicated that CD4+ CTLs may be involved, given their proinflammatory and chemotactic characteristics (20). Stimulation experiments confirmed the important role of Th1 cells and CTLs in the pathogenesis of GO, but Th17 cells may need to be studied further.

Hypoxia was enriched as the key biological pathway of the pre-GO state and was upregulated in the pre-GO state compared with both the GO and GH states. CD4+ T-cell differentiation and function, especially in Th1 and Th17 cells, are well known to be governed by hypoxia (30-32). Low oxygen levels were also reported to promote the cytotoxicity of CD8+ T-cells (33). In our study, hypoxia promoted Th1/Th17 differentiation and CD4+ CTL differentiation under different stimulation conditions, which indicated the potential effect of hypoxia on effector CD4+ T-cell differentiation in GO. In addition, the hypoxia-induced differentiation of CD4+ CTLs by antigen-specific stimulation highlights the important role of hypoxia in the antigen-derived process of GO pathology, which needs to be fully explored.

There are several limitations of this study. First, the mRNA and DNA methylation results do not precisely follow the “hypermethylation corresponds to low expression” and “hypomethylation corresponds to overexpression” patterns reported in other studies (43, 44), which indicates the complexity of the regulatory effect of methylation on gene expression and needs to be studied further. Second, owing to sampling limitations, we did not perform in vitro experiments on the PBMCs of pre-GO patients. Third, as this was a single-center study, the sample size was limited and unbalanced, so we will conduct multicenter studies with larger cohorts in the future.

## Conclusions

In conclusion, we reported that the pre-GO state significantly differed from the GH and GO states according to the integrated analysis of mRNA expression and DNA methylation. The pre-GO state is defined as the 6-month incubation period of GO and is characterized by hypoxia, which may promote the differentiation of effector CD4+ T-cells. These results not only confirm the possibility of early intervention for GO but also provide insight into the pathogenesis of and potential approaches to prevent GO.

## Data Availability

The datasets generated and analyzed during the current study are available in the Sequence Read Archive data repository (https://www.ncbi.nlm.nih.gov/sra, record number: PRJNA1137109) and Gene Expression Omnibus data repository (https://www.ncbi.nlm.nih.gov/geo/, record number: GSE272832) or in the data repositories listed in References.
